# Chemotherapy-Induced Cell-Surface GRP78 Expression as a Prognostic Marker for Invasiveness of Metastatic Triple-Negative Breast Cancer

**DOI:** 10.1007/s10439-024-03673-z

**Published:** 2025-01-05

**Authors:** Martha B. Alvarez-Elizondo, Annat Raiter, Rinat Yerushalmi, Daphne Weihs

**Affiliations:** 1 Faculty of Biomedical Engineering, Technion-Israel Institute of Technology, 3200003 Haifa, Israel; 2 Felsenstein Medical Research Center, 49100 Petach Tikva, Israel; 3 Faculty of Medicine, Tel Aviv University, Tel Aviv, Israel; 4 Department of Mathematics and Statistics, Faculty of Science, University of Hasselt, 3590 Diepenbeek, Belgium

**Keywords:** Cancer metastasis, Cell invasiveness, Mechanobiology, Doxorubicin, Chemotherapy resistance

## Abstract

Metastasis remains the leading cause (90%) of cancer-related mortality, especially in metastatic triple-negative breast cancer (TNBC). Improved understanding of molecular drivers in the metastatic cascade is crucial, to find accurate prognostic markers for invasiveness after chemotherapy treatment. Current breast cancer chemotherapy treatments include doxorubicin and paclitaxel, inducing various effects, such as the unfolded protein response (UPR). The key regulator of the UPR is the 78-kDa glucose-regulated protein (GRP78), which is associated with metastatic disease, although, its expression level in the context of invasiveness is still controversial. We evaluate doxorubicin effects on TNBC cells, identifying GRP78 subpopulations linked to invasiveness. Specifically, we evaluate the motility and invasiveness of GRP78 positive vs. negative cell subpopulations by two different assays: the in vitro Boyden chamber migration assay and our innovative, rapid (2–3 h) clinically relevant, mechanobiology-based invasiveness assay. We validate chemotherapy-induced increase in the subpopulation of cell-surface GRP78(+) in two human, metastatic TNBC cell lines: MDA-MB-231 and MDA-MB-468. The GRP78(+) cell subpopulation exhibits reduced invasiveness and metastatic potential, as compared to whole-population control and to the GRP78(−) cell subpopulation, which are both highly invasive. Thus, using our innovative, clinically relevant assay, we rapidly (on clinical timescale) validate that GRP78(−) cells are likely linked with invasiveness, yet also demonstrate that combination of the GRP78(+) and GRP78(−) cells could increase the overall metastatic potential. Our results and approach could provide patient-personalized predictive marker for the expected benefits of chemotherapy in TNBC patients and potentially reveal non-responders to chemotherapy while also allowing evaluation of the clinical risk for metastasis.

**Supplementary Information:**

The online version contains supplementary material available at 10.1007/s10439-024-03673-z.

## Introduction

Triple-negative breast cancer (TNBC) [1, 2] is a heterogeneous subtype of breast cancer, accounting for 10–20% of breast cancers. TNBC is aggressive with a high recurrence rate (recurrence within 3 years after diagnosis) and short survival times. The vast majority of TNBCs are highly invasive with high rates of distant metastasis rates and bad prognoses, and are especially prevalent in young patients. Chemoresistance and metastases remain the main obstacles in cancer therapy, especially in TNBC, and therefore identifying novel prognostic/predictive tumor markers is critical for improving cancer-treatment outcomes. Such a potential predictive marker is the 78-kDa glucose-regulated protein (GRP78), a key regulator of the endoplasmic reticulum (ER) stress response that protects cells against stress-induced apoptosis. Overexpression of GRP78 in cancer cells is expected, as a result of the inherently stressful conditions in the tumor microenvironment that include glucose starvation, lactic acidosis, and hypoxia [3]. GRP78, a chaperone in the cytoplasm of cancer cells, is overexpressed during ER stress and translocates to the cell-surface where it acts as a receptor [3, 4]. Overexpression of cell-surface GRP78 has been identified in various cancer types, including prostate [5], gastric [6], ovarian [7], thyroid [8], skin [9], and breast cancer making GRP78 a promising target for cancer-treatment. Chemotherapy is known to trigger ER stress and to promote cell-surface expression of GRP78 through the unfolded protein response (UPR) pathway, thereby preventing progression of tumor metastasis [10]. Aptly, we have previously demonstrated that doxorubicin-induced GRP78 translocation to the cell-surface of doxorubicin-treated breast cancer cells facilitates cell-tagging for apoptosis [11], potentially improving treatment outcomes for those cells. In the current study, we revealed the connection between chemotherapy-induced GRP78 expression and cancer cell invasiveness. As we had previously shown, chemotherapy may in fact enhance metastasis [12, 13], and therefore, providing a potential prognostic marker for treatment effectiveness as well as for metastasis could affect disease management and personalized application of treatments.

Various signaling pathways that regulate apoptosis, cell invasion, and metastasis [14, 15] are associated with GRP78 cell localization. It is thus not surprising that GRP78 overexpression is correlated with malignant, metastatic, and chemoresistant cancer cells [16]. GRP78 overexpression may also be associated with the epithelial-to-mesenchymal transition of tumor cells [17] that could support metastatic invasion, yet few studies have assessed the connection of GRP78 expression with the invasive capacity [14, 15]. In vitro studies show that GRP78 protects cells from chemotherapeutic agents [18], which agrees with observed chemoresistance to doxorubicin that results in the 5-year recurrence rate of 50% in stage II and III TNBC [19]. Aptly, clinical studies have shown that GRP78 cell-surface expression is correlated with poor prognosis in breast, liver, prostate, colon, and gastric cancers [20, 21]; that is due to high pathological grade, recurrence, and chemotherapy resistance. However, contradictory results-related GRP78 expression in breast cancer cells to good prognosis [22]. Those contradictory findings highlight the need to understand how GRP78 cell-surface expression relates to metastatic invasion of cancer cells, while potentially providing novel predictive markers for prognosis and treatment outcomes.

Here, we evaluate the effect of doxorubicin on GRP78 cell-surface expression and on cell migration and invasion capabilities in two human, metastatic TNBC cell lines, i.e., MDA-MB-231 and MDA-MB-468. Specifically, we evaluate effects of doxorubicin treatment on the expression of cell-surface GRP78, showing variations in the responses of the two evaluated TNBC cell lines alongside effects on the mobility of the cells, as determined via transwell assays. In addition, we use our clinically relevant, gel-based mechanobiological invasiveness assay [23–25] to evaluate changes in invasiveness and metastatic potential as a function of the GRP78 expression. We demonstrate differences between the mobility and invasiveness of the cells following doxorubicin treatment that likely result from reduced mobility and invasiveness GRP78(+) cell subpopulation. Confirming the direct connection between the metastatic potential of TNBC cells and the cell-surface GRP78 expression can provide insights to new and more effective treatments and reveal predictive factors for chemo-responsiveness in the context of TNBC invasiveness.

## Materials and Methods

### Cells

Two human, TNBC cell lines were used in these experiments: MDA-MB-231 and MDA-MB-468 (respectively HTB-26 and HTB-132, both from American Tissue Culture Collection, ATCC, Manassas, VA). Both breast cancer cell lines were originally collected from pleural effusion and are, respectively, high and low metastatic potential. Cell cultures were maintained at 37 °C, in a humidified, 5% CO_2_ incubator in Dulbecco’s modified Eagle’s medium (DMEM, Gibco, Carlsbad, CA) supplemented with 10% fetal bovine serum (FBS, Hyclone Thermo Fisher Scientific, Waltham, MA) and 1 vol% each of l-glutamine, sodium pyruvate, and penicillin-streptomycin (all from Biological Industries, Kibbutz Beit Haemek, Israel). Cells were used at passages 10–30 from ATCC stock.

### Expression Levels of GRP78

The expression of surface GRP78 in the cell lines was increased using doxorubicin (Ebewe Pharma, Unterach am Attersee, Austria). Fluorescence-activated cell sorting (FACS) was used to determine the percentage of cells expressing surface GRP78 following 24-h incubation with doxorubicin concentrations of 0.01, 1, and 10 μg/ml (*n* = 9) and cell viability was quantified via counting after Trypan Blue staining, as compared to untreated control. In short, cells from both lines (2.5 × 10 [6] cells/dish) were detached using cold phosphate buffered saline (PBS) and 0.5 × 10 [6] cells transferred to FACS tubes for staining. After centrifugation (1200 rpm for 10 min), cells were resuspended in 50 μl PBS supplemented with 5% FBS (Gibco, Invitrogen) and 0.1% NaN_3_ (Sigma-Aldrich, KGaA, Darmstadt, Germany). To detect cell-surface GRP78, monoclonal anti-GRP78 antibody (DyLight^®^ 488, Abcam, Cambridge, MA) was added to tubes at a concentration of 2 μg/ml and incubated for 1 h at 4 °C. After washing, cells were suspended in 400 μl PBS and subjected for flow cytometry (Coulter Navios flow cytometer, Indianapolis, IN, USA). To ascertain nonspecific binding, isotype control was determined using IgG 2a DyLight^®^ 488 antibody. Gated stained live cells were assessed using the FACS Kaluza software (Beckman Coulter Life Sciences, Indianapolis, IN). A doxorubicin concentration of 1 μg/ml (incubation for 24 h) was selected for the subsequent experiments, to maximize GRP78 expression while maintaining > 90% cell viability.

### Collection of GRP78 Cell Subpopulations

**Fig. 1:**
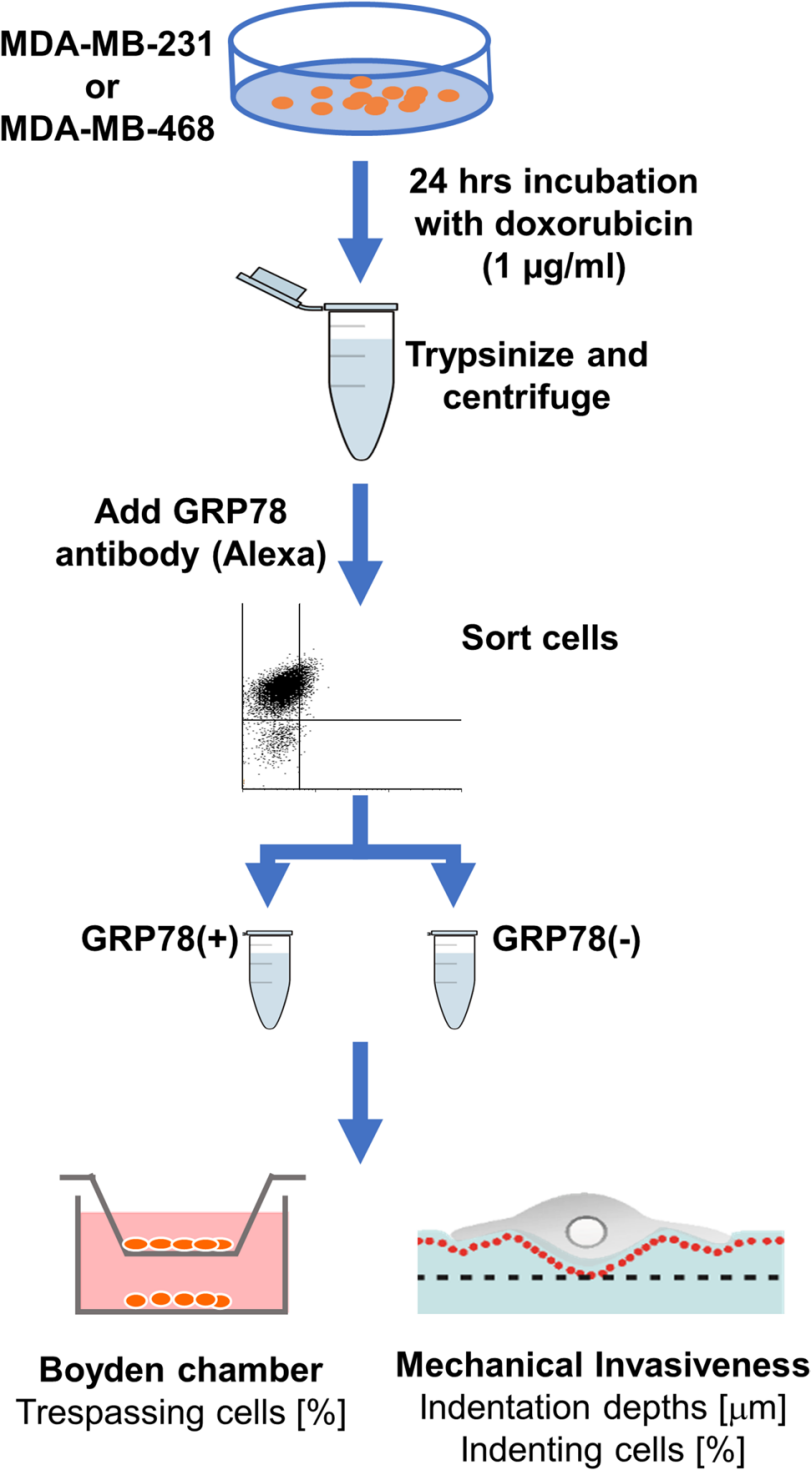
The experimental protocol: Evaluation of the invasiveness of TNBC cells subpopulations induced by chemotherapy. MDA-MB-231 and MDA-MB-468 cell lines were incubated with 1 µg/ml doxorubicin for 24 h. Cells were then trypsinized and stained with monoclonal GRP78 antibody. Cells with positive and negative GRP78 expression were collected using FACS. The invasive capacity of those subpopulations was evaluated using Boyden chambers and our mechanical invasiveness assays. The obtained values were compared to the unsorted (whole-population) control for both assays

GRP78 positive- and negative-expression subpopulations were generated in both cell lines via 24-h treatment with 1 μg/ml doxorubicin and were sorted and collected using FACS. In short, cells (10 [6] cells/0.5 ml) were stained with 2 μg/ml monoclonal anti-GRP78 antibody and incubated for 1 h at 4 °C. Cells were sorted in a FACSAria II machine (BD Life Sciences, San Jose, CA), using a 488 nm blue laser with a 530/30 nm bandpass filter; the sorting was performed at the Lorry I. Lokey Interdisciplinary Center for Life Sciences and Engineering at the Technion-IIT. We captured 10,000–20,000 events (cells) and data were analyzed with the BD FACSDiva Software. Unstained, same-passage cells were similarly run through the FACS to control for any flow and staining effects and were used as the whole-population control. The dot-plots were used to gate forward-scatter and side scatter bounds to exclude any debris, dead cells, cell-clumps or doublets [25]. Collected cells with GRP78 positive- and negative-expression were used for further testing, and were compared to whole-population controls (Fig. 1).

### Transwell Migration

We performed a migration assay using 8 µm pore Boyden chamber membranes in 24-well plates (Greiner Bio-One, Frickenhausen, Germany) coated with 100 µl Matrigel Basement Membrane Matrix (BD Biosciences, San Jose, CA). The MDA-MB-231 and MDA-MB-468 breast cancer cells were suspended using 0.25% Trypsin-EDTA solution (Biological Industries, Israel). Cells after treatment with 1 µg/ml Doxorubicin or untreated control cells were seeded at 2 × 10 [5] cells in 100 µl of serum-free DMEM (migration buffer) on top of the transwell membranes (Fig. 1). The chemo-attractant added to the lower chamber was 600 µl of DMEM containing 10% FBS. Following incubation at 37 °C for 12–16 h, cells that had migrated into the lower chamber were fixed with paraformaldehyde 4% and methanol 100% (both Merck KGaA, Darmstadt, Germany), stained with Giemsa (Merck, Rahway, NJ) for 15 min, and washed twice with PBS. The cells that had migrated were counted using an inverted microscope.

### Gel Preparation

Polyacrylamide gels with fluorescent microparticles embedded at their surface, as fiducial markers for the gel-surface location, were prepared following our previously established protocols [24, 26–29]. In short, gels were prepared on #5 thickness (0.5–0.6 mm) glass coverslips, 30 mm in diameter (Menzel, Germany), which were first hydroxylized using 0.1 M NaOH (Sigma-Aldrich, St. Louis, MO), activated with 3-aminopropyltrimethoxysilan (Sigma-Aldrich, St. Louis, MO), and fixed with glutaraldehyde. Gel-monomer solutions of 34 μl of 40 vol% acryl and 3.8 μl of 2 vol% BIS acrylamide (both from Bio-Rad, Hercules, CA) in 203 µl of distilled water were prepared on ice; this recipe produced a gel with Young’s modulus of 2.4 ± 0.14 kPa, as previously determined at our laboratory using shear rheometry [23, 28, 30]. Gelation was initiated with 1:200 vol. ammonium persulfate (APS) and catalyzed with 1:500 vol. tetramethylethylenediamine, TEMED (both from Sigma, St Louis, MO). Fluorescent polystyrene particles, 200 nm diameter (Molecular Probes, Invitrogen Life Technologies, Carlsbad, CA) were added to gel solutions, which were placed on the glass within 10 × 10 mm [2] gene-frames (ABgene Thermo Scientific, Waltham, MA). Gelation was performed at 2 °C under 300×*g* centrifugation for 30 min, to bring to particles the gel-surface. Finally, the gel-surface was activated with Sulfo-SANPAH (Pierce, Thermo Scientific, Waltham, MA) and coated with rat tail, collagen type I (Sigma, St Louis, MO) to facilitate cell adhesion. Gels were kept at 4 °C for up to 2 weeks.

### Imaging and Mechanical Invasiveness Gel-Assay

Each of the sorted subpopulations (GRP78(±) and unsorted flow-control) of MDA-MB-231 and MDA-MB-468 cells (Fig. 1) were seeded (10 [5] cells in 2 ml) on separate 2.4 kPa gels and were imaged 1 h after seeding to allow cell adhesion. Using our combined on-stage and microscope incubators (Life Imaging Services, Switzerland) cell culture conditions, 37 °C, 5% CO_2_, and high humidity (90%), were maintained throughout the experiment. Imaging was performed with an inverted, epifluorescence Olympus IX81 microscope (Olympus Japan), using a long working-distance, 60×/0.7 NA, differential interference contrast (DIC, Nomarsky optics) air-immersion objective lens. Images were captured using an XR Mega-10AWCL camera (Stanford Photonics, Palo Alto, CA) with a final magnification of 107.8 nm/pixel.

Three different experiments were performed for each subpopulation for each cell line, 1 h after cell-seeding. We imaged at least 15 randomly chosen fields-of-view on each gel of the MDA-MB-231 and MDA-MB-468 cell lines, providing, respectively, 165/438 (indenting/total imaged cells) and 152/677 cells. In each fields-of-view, the following images were collected according to our established assay [24, 27–29, 31, 32]: (a) a DIC image of the cell on the gel, (b) a fluorescence image of the particles at the gel-surface height, and (c) when a cell indented the gel, a fluorescence image at the lowest focal depth where the surface-particles were in focus. The percentage of indenting cells was determined for each subpopulation and for the unsorted (whole-population) control; to compensate for variable and small numbers of cells in each field-of-view, the percentage of indenting cells was pooled for all the fields-of-view in each experiment. The indentation depth was calculated from the difference between the focal heights of the gel-surface and of the lowest plane where the displaced gel-surface beads are in focus i.e., the difference between the focal heights of the two fluorescence images.

### Data Analysis and Statistics

A *T* test was used to compare the GRP78 expression following exposure to different Doxorubicin concentrations, *p* < 0.01 was considered significant. To compare the different cell subpopulations, we have used a two-sample Kolmogorov–Smirnov test with a confidence level of 95% (*p* < 0.001); this test compares different samples and is also sensitive to the normality of their distributions. Results are presented as mean ± standard error of the mean, unless otherwise specified.

## Results

**Fig. 2:**
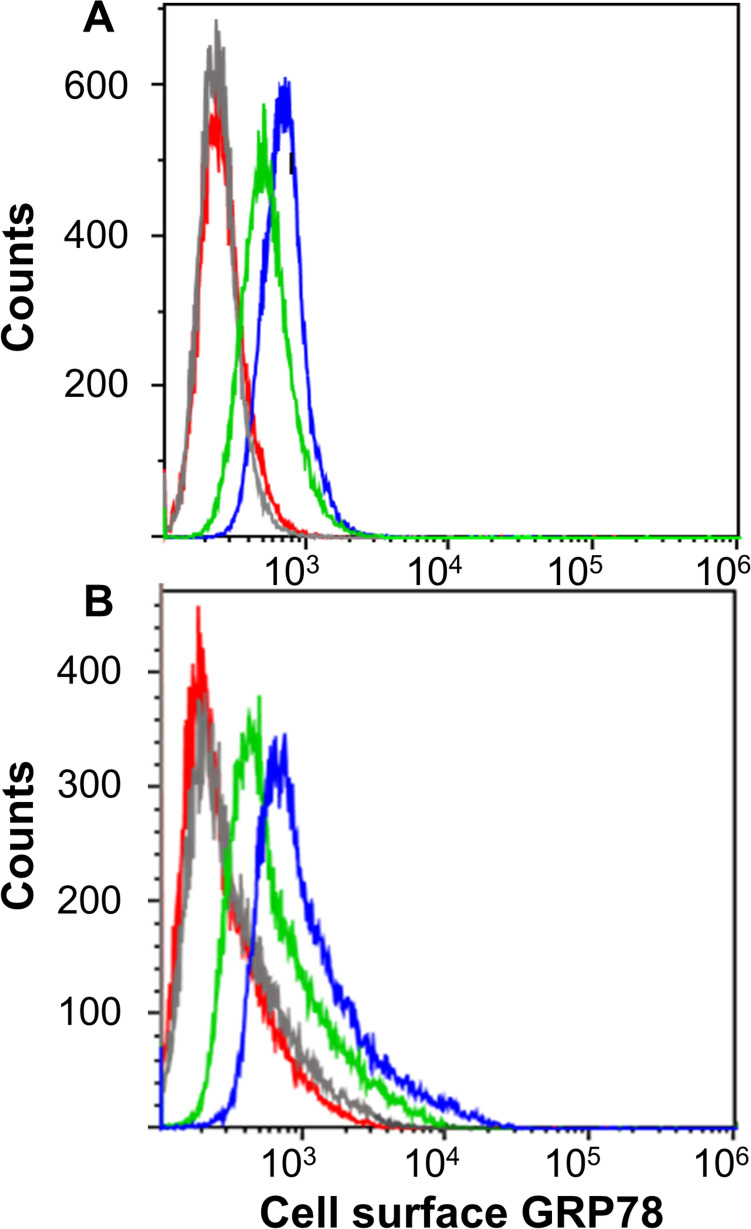
Cell-surface expression of GRP78 is increased in TNBC cells under doxorubicin treatment, as evaluated by fluorescence-activated cell sorting (FACS) analysis. **A** MDA-MB-468 and **B** MDA-MB-231 cell lines (*n* = 9 for both), were treated with increasing Doxorubicin concentrations of 0.1 µg/ml (red), 1 µg/ml (green) and 10 µg/ml (blue) as compared to Control (gray). Percent of GRP78 expressing cells provided in Table S1

Doxorubicin treatment increased cell-surface expression of GRP78 in TNBC cells (Fig. 2 and Table S1). We evaluated the concentration-dependent effects of doxorubicin on the expression of cell-surface GRP78 in two TNBC cell lines: MDA-MB-231 and MDA-MB-468 (Fig. 1). The TNBC cell lines were incubated for 24 h with four different concentrations of doxorubicin (i.e., 0, 0.1, 1 and 10 µg/ml), and the percentage of cells expressing GRP78 was determined by FACS (Fig. 2). The baseline-untreated percentage of cell-surface GRP78 was similar for both cell lines (Table S1) and increased similarly and non-significantly under 0.1 µg/ml doxorubicin. We observed a statistically significant increase in GRP78 expression in both TNBC cell lines following treatment with 1 and 10 µg/ml doxorubicin, with a more significant increase for the MDA-MB-231 cells as compared to the MDA-MB-468 cells. In all subsequent experiments, we used the doxorubicin concentration of 1 µg/ml, as it significantly increased the percentage of GRP78(+) cells while maintaining > 90% viability.

**Fig. 3:**
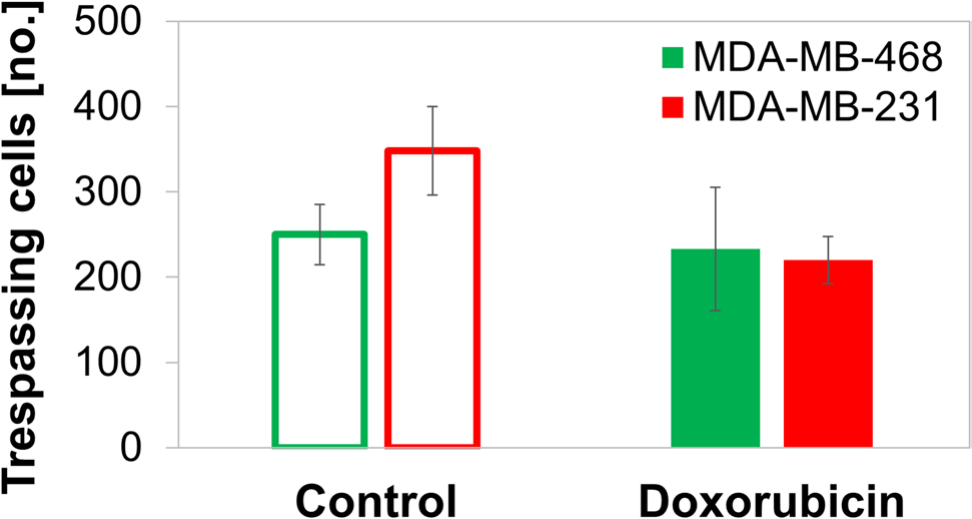
Migration of MDA-MB-231 (red) and MDA-MB-468 (green) cells over 12–16 h through 8-µm-pore Boyden chambers with membranes. Cells treated for 24-h treatment with 1 µg/ml doxorubicin are compared to untreated, control cells. Error bars are standard deviations (*n* = 2)

Doxorubicin-induced GRP78 expression reduced the overall mobility of the TNBC cell samples. Using the TNBC cells with significantly increased cell-surface GRP78 expression following treatment with 1 µg/ml doxorubicin for 24 h, we evaluated the corresponding changes in invasive capacity of those cells (Fig. 1). To evaluate the motility and invasiveness, we compare results from two assays: transwell, Boyden chamber assays and mechanical invasiveness, gel-indentation assays. Cell mobility through 8 µm pore Boyden chamber membranes demonstrated reduction of invasiveness for the MDA-MB-231 cells, yet little effect on the MDA-MB-468 cells (Fig. 3). Specifically, doxorubicin treatment reduced the number of MDA-MB-231 cell that transmigrated the Boyden chamber membrane by 36.8%, while the reduction was only 6% in the MDA-MB-468. Hence, TNBC cell-mobility is reduced in varying degrees under doxorubicin treatment.

**Fig. 4:**
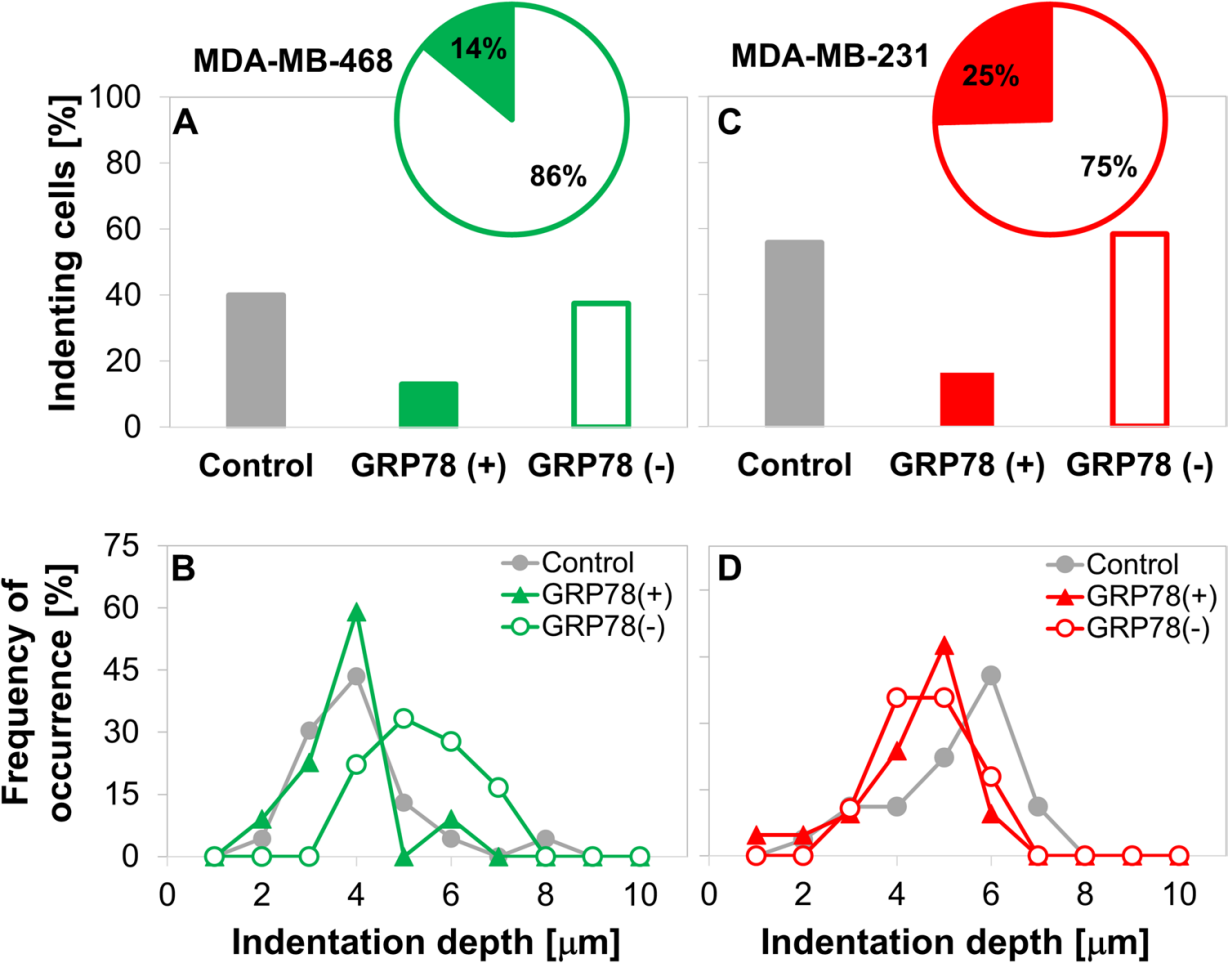
Mechanical invasiveness assay results indicating invasively indenting cell subpopulations dependence on GRP78 expression in the percentage of indenting **A** MDA-MB-468 and **C** MDA-MB-231 cells is higher for GRP78(−) cells. Insets provide the GRP78 expression levels. The distributions of the attained indentation depths in **B** MDA-MB-468 and **D** MDA-MB-231 cells vary differently with GRP78 expression

The doxorubicin-induced, GRP78 cell-surface expressing, GRP78(+) subpopulation in the TNBC cells demonstrates notably lower invasive capacity relative to GRP78(−) subpopulation (Fig. 4). Using our mechanobiology-based invasiveness assay [23–25], we evaluate the mechanical invasiveness of the cells, by determining the amounts of invasively indenting cells and their attained depths (Fig. 1). Figure 4A, C shows that the GRP78(−) cells are more invasive in both TNBC cells, producing the invasiveness level observed in the whole-population (doxorubicin-treated and unsorted) control; the percentage of cells that were GRP78(+) after 1 µg/ml doxorubicin treatment was higher in the MDA-MB-231 than the MDA-MB-468 cells (insets of Fig. 4A, C). The distributions of indentation depths for both cell lines were Gaussian-like, and were 

higher for the unsorted, whole-population control MDA-MB-231 cells as compared to the MDA-MB-468 controls (Fig. 4B, D). Interestingly, the GRP78(−) subpopulation of the MDA-MB-468 cells attained deeper depths than the unsorted, whole-population control for that cell line, on the scale of the higher-invasiveness MDA-MB-231 cells. It is, however, a combination of the percent of indenting cells and their attained depth that determines the invasiveness of the cell subpopulation [23, 25].

**Fig. 5:**
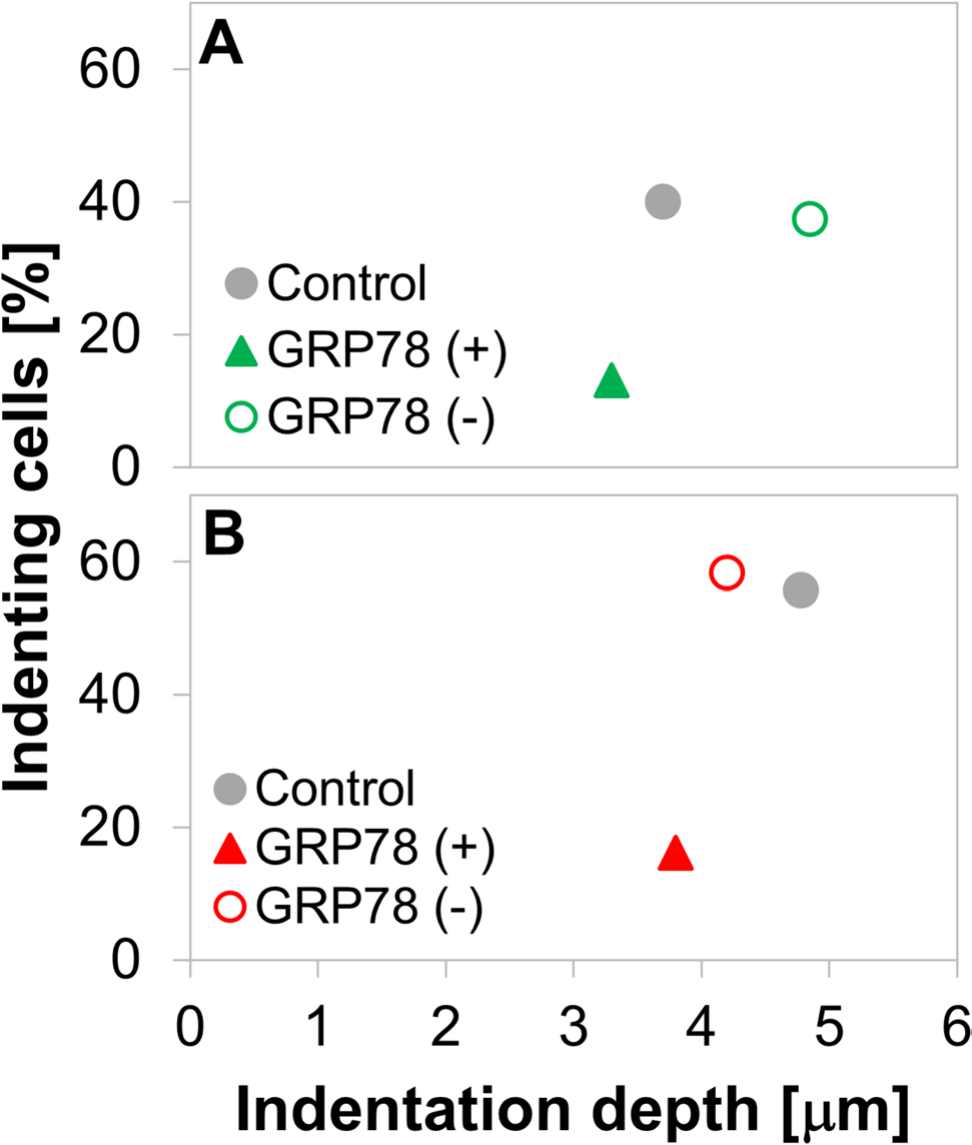
Mechanical invasiveness of the **A** MDA-MB-468, **B** MDA-MB-231 TNBC cell lines. The GRP78(+) cells (full triangles) are significantly less invasive than the GRP78(−) cell subpopulation (empty circles) and the control cells (full circles) in both TNBC cell lines

The mechanical invasiveness of GRP78(+) cells was low in both evaluated TNBC cell lines (Fig. 5). The MDA-MB-231 cell line, unsurprisingly, demonstrated higher-invasiveness relative to the MDA-MB-468 cells, and importantly a larger GRP78(+) subpopulation was induced by the doxorubicin treatment. The differences in invasiveness between the cell subpopulations in both TNBC cell lines are highlighted when combining the two measures of indentation depth and percentage of indenting cells (Fig. 5). The small differences in the percent of indenting cells between the GRP78(−) and unsorted control (i.e., lower in the MDA-MB-231 GRP78(−) cells than the control and higher in the MDA-MB-468) were offset by the changes in the average indentation depth (higher in the MDA-MB-231 and lower in the MDA-MB-468 cells), likely resulting in similar invasiveness. In contrast, the GRP78(+) subpopulation was significantly reduced in both measures. We thus observe, in both cell lines, that the mechanical invasiveness of the GRP78(−) subpopulations and the whole-population controls are similar and are both significantly (*p* < 0.001) more invasive than the GRP78(+) cell subpopulations.

## Discussion

Cell invasion through surrounding tissue is one of the first steps of the tumor metastatic cascade, which we show is directly affected by the GRP78 cell-surface expression. Regarding controversial literature on the significance of cell-surface GRP78 expression in determining metastatic potential, our experiments confirm that doxorubicin treatment produces a TNBC cell subpopulation that express cell-surface GRP78 (i.e., GRP78(+)), and those GRP78(+) cells are less invasive than the GRP78(−) cell subpopulation. As a result, the doxorubicin treatment may reduce the overall migratory capacity and invasiveness of the TNBC cell samples.

Our results demonstrate a significant increase in amounts of GRP78(+) TNBC cells in response to doxorubicin in both evaluated metastatic TNBC cell lines. This is consistent with the increase in GRP78 cell-surface expression that we had previously observed in tumor samples from patients that had undergone systemic chemotherapy [20]; no significant differences in cytoplasmic GRP78 were detected between those groups. For TNBC, the most aggressive and deadly breast-cancer subtype with high rates of recurrence and poor survival, doxorubicin and paclitaxel are currently the standard, prescribed chemotherapy. The mechanism of action of those chemotherapies is, in part, to induce the UPR through the ER stress pathway in cancer cells [18, 33, 34]. The UPR enhances the degradation of misfolded proteins and increases the folding capacity of the ER by recruiting GRP78 to the accumulating misfolded proteins acting as a chaperone. As a result of ER stress-induced by chemotherapy, GRP78 translocates to the cell-surface of cancer cells thereby promoting therapeutic resistance as a primary effect and by promoting a signaling cascade for cell apoptosis as a result of prolonged ER stress [35–37]. Increased expression of cell-surface GRP78, affects the migratory and invasive capacities of the cells [38].

We show that the sample mobility [39] and its invasiveness reduce following doxorubicin treatment, likely due to the increase in the GRP78(+) cell subpopulation, which we show is less invasive (Fig. 5). The doxorubicin-induced increase in GRP78(+) subpopulation and ensuing reduction in invasiveness was more pronounced in the higher-invasiveness MDA-MB-231 cell line as compared to the MDA-MB-468 cells although the latter are more proliferative [40]. We previously observed similar relative responses in the same two TNBC cell lines also to paclitaxel treatment [29]. Importantly, the higher-invasiveness of the GRP78(−) subpopulation indicates its significant contribution to the invasiveness and the metastatic potential of TNBC cells. Thus, our results indicate that cell-surface GRP78 expression in TNBC is linked to reduced migratory capacity and invasiveness. In parallel, we note that while doxorubicin produced a less invasive cell-surface GRP78(+) cell subpopulation, it is important to emphasize that the cell samples remain highly mobile and invasive, which may reconcile controversies in the clinical literature as to the correlation of GRP78 expression and invasiveness.

We show that the cell-surface GRP78 expression in TNBC cells is associated with reduced migratory capacity and invasiveness, which is expected to decrease the likelihood for metastasis and improved cancer-patient prognosis. This observation agrees with previous clinical investigations that have demonstrated the association of cell-surface GRP78 expression after doxorubicin treatment with good clinical prognosis in breast and other cancer types [22, 41]. While GRP78 expression (i.e., existence of GRP78(+) cells) correlated with favorable prognosis in lung cancer [42], and increased 5-year survival in colon cancer [43], in some cases, conflicting results with negative clinical outcomes [44, 45] were observed in breast cancer [46], gastric cancer [47], pancreatic cancer [48] and liver cancer [49]. The conflicting clinical outcomes across studies may result from the cancer cell origin, as GRP78 has been demonstrated to have distinct roles in different cancer types [50]. Concurrently, within the same cancer type, the UPR pathway can be either cytoprotective or disruptive to the metastatic process, depending on its activation level [51].

CREB3L1 is a transcription factor of GRP78 [52], and significant increase in expression of both cell-surface GRP78 and CREB3L1 was demonstrated in response to doxorubicin, specifically also in the same TNBC cell lines [39]. Interestingly, CREB3L1 down regulation decreased GRP78 expression resulting in suppression of the metastasis cascade, while CREB3L1 knockout revoked the metastatic suppression, inhibited GRP78 expression in the MDA-MB-231 cell line, and induced a massive metastatic profile in mouse-models despite chemotherapy [39]. Our results correspondingly demonstrate that the GRP78(−) subpopulation is the most invasive. In the current work, we have demonstrated that our novel mechanobiology-based assay, which is sensitive to cell subpopulations [24, 25], can rapidly (within 2–3 h) provide the invasiveness level of a sample, associated with the GRP78 expression as a prognostic marker for the metastatic likelihood following chemotherapy.

We propose that the clinical outcomes in cancer may be controlled and also identified through changes in the cell-surface GRP78 expression in different cell subpopulations and the resulting effect on invasiveness; those can produce phenotypes with varying invasiveness and change the overall invasiveness of the sample. It is important to note that while the GRP78(+) subpopulation demonstrated reduced invasiveness in the evaluated TNBC cell lines, it did not become non-invasive. Therefore, the overall invasiveness of the sample, while potentially reduced following doxorubicin treatment, can still result in metastases, especially if further modifications occur that can support metastasis. We have previously demonstrated, in experiments and computational modeling, that cancer cell proximity can increase the invasiveness of individual closely adjacent cells, effectively increasing the overall sample invasiveness and metastatic potential [29, 30, 53, 54], as is also likely the case with the different GRP78 subpopulations in a heterogeneous sample. In addition, the prolonged ER stress in the TNBC cells could potentially increase the invasiveness of the cells. For example, GRP78(+) breast cancer cell subpopulations exhibit elevated expression of stem-cell-related genes with increased aggressiveness and tumorigenicity in vitro and in vivo [4]; breast-cancer stem cells have been shown to be more invasive than other cancer cell subpopulations [25]. Similarly, in hepatoma cells, the insulin-like growth factor I enhanced cell-surface GRP78(+) expression, and resulted in a malignant cell phenotype with increased proliferative and migratory capacities [55]. Concurrently, when GRP78 is induced by chemotherapy, its over-expression can inhibit drug effectiveness and cause chemoresistance [22, 56], specifically in TNBC cells [57]. Taxol treatment has aptly induced metastasis-supporting terraforming of the extracellular matrix at distant sites, via extracellular vesicles and partially supported by T-cells, even when breast cancer cells were not present in the host [12, 13]. Thus, contradictory results that correlate GRP78(+) cells with increased invasion and metastasis could result from combined interactions of GRP78(+) and GRP78(−) cell subpopulations alongside effects of extra mutations that together can increase the overall sample invasiveness and likelihood for metastasis.

In conclusion, our study demonstrates that cell-surface GRP78 expression in TNBC, e.g., induced by chemotherapy, is linked to reduced migratory capacity and invasiveness, potentially decreasing the likelihood of metastasis and improving patient prognosis. We have demonstrated that presence of cell-surface GRP78(+) cell subpopulations is a potential predictor of the metastasis reducing effects of doxorubicin treatment. However, as the highly invasive GRP78(−) subpopulation remains large, and the GRP78(+) subpopulation is still invasive (albeit lower level), the overall sample invasiveness may remain high and may still support metastases formation contributing to the heterogeneity of TNBC samples. Nevertheless, the cell-surface GRP78 expression in TNBC cells (i.e., existence or increase of the GRP78(+) subpopulation) may be a marker for good prognosis following chemotherapy treatment. Given the importance of finding faithful biomarkers to prognose the metastatic potential in the aggressive TNBC disease, we supported our results using our novel mechanobiology methodology to accurately quantify cell invasiveness. While the combined effects of different cancer cell subpopulations expressing factors associated with invasiveness (such as GRP78 subpopulations) remains elusive due to their complex combined interactions, our clinically relevant, mechanobiological methodology rapidly answers the important question of—is the sample less or more invasive following chemotherapy?—and could therefore provide accurate prognosis and direct choice of effective treatment strategies.

## Supplementary Information

Below is the link to the electronic supplementary material.

Supplementary file1 (DOCX 15 KB)
