# Supplementary material for: Chemotherapy-Induced Cell-Surface GRP78 Expression as a Prognostic Marker for Invasiveness of Metastatic Triple-Negative Breast Cancer
Source: Ann Biomed Eng. 2025 Jan 5;53(4):881–90. doi: 10.1007/s10439-024-03673-z (PMC11929716; doi:10.1007/s10439-024-03673-z)
Supplement: Supplementary file 1 — Supplementary file1 (DOCX 15 KB) [file 10439_2024_3673_MOESM1_ESM.docx]

**Electronic Supplementary Materials**

**Table S1**. Percent of cells expressing GRP78 following treatment with varying doxorubicin concentrations

| Cell line | Doxorubicin concentration [µg/ml]^a^ | | | |
| --- | --- | --- | --- | --- |
|  | 0 (control) | 0.1 | 1 | 10 |
| MDA-MB-468 | 7.7 ± 4.6 | 9.7 ± 3.3  (p=0.265) | 14.6 ± 3.7  (p=0.0007) | 30.8 ± 6.6  (p=1.627x10^-10^) |
| MDA-MB-231 | 8.6 ± 6.4 | 9.6 ± 3.9  (p=0.675) | 25.4 ± 10  (p=0.0001) | 45.6 ± 12.4  (p=1.33x10^-8^) |

^a^ Values are presented as mean ± standard deviation and values in parenthesis are the p-value relative to control
